# The Assessment of Knowledge and Early Management of Acute Pancreatitis Among Residents

**DOI:** 10.7759/cureus.4389

**Published:** 2019-04-05

**Authors:** Asif Mehmood, Waqas Ullah, Vincent Chan, Daniel Ringold

**Affiliations:** 1 Internal Medicine, Geisinger Medical Center, Danville, USA; 2 Internal Medicine, Abington Hospital-Jefferson Health, Abington, USA; 3 Internal Medicine, Abington Hospital - Jefferson Health, Abington, USA

**Keywords:** acute pancreatitis, pancreatic inflammation

## Abstract

There has been a rapid increase in the incidence of acute pancreatitis (AP) and associated mortality. This mortality is even higher in patients having severe disease (about 30% in contrast to 10% in mild AP). Some of the factors which have proven to lower the mortality are early feeding rather than keeping patient nil per oral (NPO), and aggressive intravenous fluid replacement therapy, especially during the first 12-24 hours. in our study, we investigated the reasons for the increase in incidence and AP-associated mortality as there was no previous study done to focus on these areas.

## Introduction

Acute pancreatitis (AP) is also known as the acute inflammatory process of the pancreas. It has an annual incidence of about 20-40 cases per 100,000 persons every year [1]. It is among one of the most common diseases of the gastrointestinal (GI) tract requiring hospitalization, and costing more than two billion dollars to the United States economy [2-3]. In our hospital, It was observed that patients with AP were not being fully resuscitated and managed according to the standard of care, 2013 American College Of Gastroenterology guidelines, which lead to the conduct of this educational study. It has been well documented in the literature that increased compliance with clinical practice guidelines results in improved patient outcomes i.e., reduced mortality, decreased length of hospital stay, and low rate of infections [4-5]. We decided to do this study to assess residents knowledge and practices about adherence to 2013 guidelines based on the American College of Gastroenterology (ACG) recommendations for the early management of AP in a community-based hospital setting so that measures can be taken to improve adherence to the standard of care guidelines for better outcomes.

## Materials and methods

This study is a cross-sectional study conducted at Abington Jefferson Health. All current internal medicine from Postgraduate Year-1 (PGY-1) to Postgraduate Year-4 (PGY-4) and surgical residents from Postgraduate Year-1 (PGY-1) to Postgraduate Year-5 (PGY-5) were included. A 16 questions data collection questionnaire form was developed based upon the 2013 guidelines by ACG about early management of AP (Appendix A). The questionnaire was created using the SurveyMonkey (SurveyMonkey Inc.; San Mateo, California, USA) tool and was sent to all participants via email.

## Results

A total of 71 participants (25 surgery, 46 internal medicine (IM)) were included in the study. Only 40 participants (56%) consisting of five in surgery and 35 in internal medicine completed the questionnaire. Out of 35 IM residents, most responses came from PGY-1 (48%, n=17), PGY-2 (25%, n=9), PGY-3 (24%,n=8) and the least from IM PGY-4 (3%, n=1) (Table 1). There were no responses from surgery PGY-1 and PGY-5 of surgery residents, while only one PGY-2 and two residents of PGY-3 and PGY-4 of a total of five surgery residents completed the questionnaire (Table 2).

**Table 1:** Frequency of responses for IM residents for each year of residency PGY= postgraduate year; IM= internal medicine.

	PGY1-IM	PGY2-IM	PGY3-IM	PGY4-IM	TOTAL IM
Number of Responses	17	9	8	1	35
Percentage	48.5%	25.7%	22.8%	2.8%	100%

**Table 2:** Frequency of responses for surgery residents for each year of residency PGY= postgraduate year

	PGY-1	PGY-2	PGY-3	PGY-4	PGY-5	TOTAL SURGERY
Number of responses	0	1	2	2	0	5
Percentage	0%	20%	40%	40%	0%	100%

**Figure 1:**
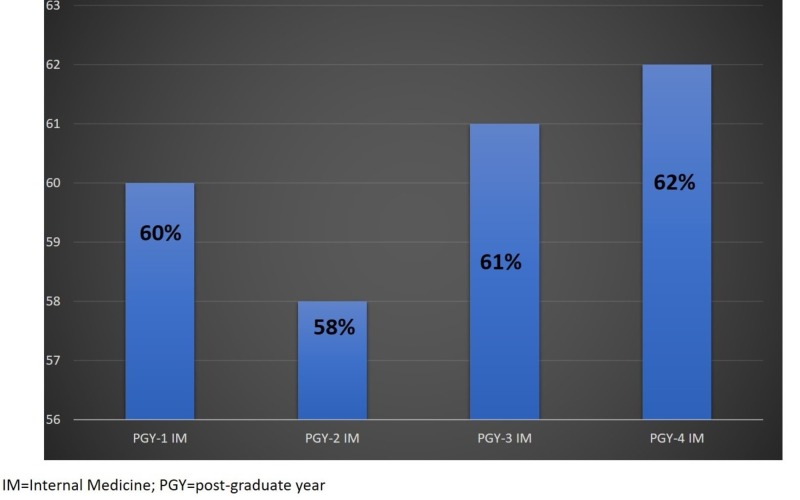
Correct answers for IM

IM PGY-3 responders, provided the most correct answers, about 61% on average for all questions in the questionnaire. While only 58% questions were answered correctly by PGY-2 and 60% questions were answered correctly by PGY-1 IM residents (Figure 1). Only one respondent from IM PGY-4 gave 62% correct answers. Only 20% (n=5/25) surgery residents responded to the survey, and they gave correct answers for about 46% questions on the questionnaire correct.

IM and surgical resident comparison for correct answers was 60% vs. 46% respectively with a low response rate from surgery residents. Residents showed lack of knowledge (<50% correct answers) specifically in questions related to diagnosis, initial assessment/risk stratification and role of endoscopic retrograde cholangiopancreatography (ERCP)/surgery. However, greater than 50% correct answers were given for fluid resuscitation, the role of antibiotics, and nutrition (Table 3).

**Table 3:** Categories of questions in IM vs surgery IM = internal medicine; AP = acute pancreatitis; ERCP = endoscopic retrograde cholangio pancreatography.

Total Responders (n=40)	Initial management of AP	Diagnosis	Initial assessment and risk stratification	Role of ERCP	Role of antibiotics in AP	Nutrition in AP	Role of surgery
TOTAL IM (n=35)	71% (n=25)	43% (n=15)	31% (n=11)	49% (n=17)	77% (n=27)	67.7%	29.7%
TOTAL SURGERY (n=5)	60% (n=3)	40% (n=2)	0%	20% (n=1)	60% (n=3)	33.3%	46.7%

## Discussion

AP is one of the common GI diseases requiring hospital admission with significant morbidity and mortality. There are many standard of care guidelines available for the management of AP issued by different gastroenterology and surgical societies. In 2010, a systematic review was done which analyzed almost 30 worldwide guidelines about AP using validated guideline scoring system. It has shown that the 2006 ACG guidelines had the highest quality scores among American based guidelines (Revised in 2013). Internationally, there are three other well-accepted guidelines available which include the British Society of Gastroenterology (BSG) guidelines, the Japan Society of Abdominal Emergency Medicine (JSAEM) guidelines, and the International Association of Pancreatology (AP) guidelines [6].

Like every disease, evidence-based treatment is essential to deliver high quality of care with better outcomes. Many studies have been done inside and outside of the United States of America which has shown poor adherence to the standard of care guidelines among physicians and surgeons [7-8]. The mortality rate in AP is influenced by many factors with various degrees including age, etiology, the extent of necrosis, and presence of multi-organ failure [9-10]. Patients managed according to standard guidelines/protocols have shown better outcome in term of complications rate, length of hospital stay, morbidity and mortality [11].

Majority of the current guidelines have consensus on measures in managing AP like early aggressive fluid resuscitation and enteral nutrition, endoscopic sphincterotomy for dilated common bile duct with impacted gallstones with impending acute cholangitis and prophylactic role of antibiotics in selected patients, which has shown better clinical outcomes [12].

There are many barriers which can lead to poor adherence to standard guidelines including physicians lack of knowledge and lack of clinical decision support tools. It has been shown that there were better clinical outcomes with increased adherence to guidelines after educating physicians and the introduction of clinical decision support tools in managing patients with AP [11-12]. It is very important that residents and physicians must have a good fund of knowledge about current guidelines for managing AP.

The limitations of this study include a single center study, low sample size, and a low response rate from surgery residents.

Residents/physicians need more education about clinical practice guidelines regarding the early management of AP. It can be achieved by arranging educational lectures and placing the summarized guidelines on hospital’s clinical decision support page. It is important to formulate an admission order set in the Electronic Medical Records system based on standardized guidelines for the early management of AP to achieve improved patient outcomes.

## Conclusions

Overall IM PGY-3 residents showed better knowledge and understanding about the standard of care guidelines for the early management of AP followed by IM PGY-1 and IM PGY-2. Residents showed a lack of knowledge in questions related to diagnosis, initial assessment/risk stratification, and the role of ERCP and surgery as compared to better performance in fluid resuscitation, the role of antibiotics, and nutrition. The low survey response rate from surgery residents prevented meaningful comparisons between the residency programs.

## Appendices

Appendix A-Early management of acute pancreatitis

1) Which early intervention decreases the mortality and morbidity in acute pancreatitis?

A) Prophylactic antibiotics B) Intravenous fluids C) Pain control D) Parenteral nutrition

2) What type of intravenous fluid is preferred in acute pancreatitis for most patients?

A) D5 ½ normal saline B) Normal saline C) Ringer’s lactate D) Albumin

3) How quickly should aggressive fluid resuscitation start?

A) Immediately B) Within 4-6 hours. C) Within 10-12 hours. D) Within 12-24 hours

4) What should be the minimal goal of intravenous resuscitation during the first 12-24 hours unless contraindications present?

A) 0.5-1 ml/kg/h B) 5-10 ml/kg/h C) 50-100 ml/kg/hr D) 100-500 ml/kg/hr

5) What is the single best estimator of intravascular volume status in acute pancreatitis?

A) Hemoglobin B) WBC count C) Heart rate D) BUN E) Systolic blood pressure

6) Which patient population needs more aggressive initial intravenous fluid therapy?

A) CHF B) Hypotensive C) Tachycardic D) Both B and C E) All of the above

7) Which patient population needs abdominal cross-sectional imaging (CT or MRI) in acute pancreatitis?

A) Gallstone pancreatitis B) Unclear diagnosis C) Fail to improve/worsen within first 48-72 hours D) Both B and C E) All

8) Which patients with acute pancreatitis should be admitted to the intensive care unit?

A) Lipase >12000 B) BUN > 30 C) WBC > 20,000 D) Organ failure E) All

9) Which patients with acute pancreatitis should undergo ERCP within 24 hours of admission?

A) Concurrent cholangitis B) Bilirubin >3 C) CBD > 6mm on USG

D) RUQ pain with abnormal LFTs E) All of the above

10) Which class of antibiotics have shown efficacy in patients having acute pancreatitis with infected necrosis?

A) Carbapenems B) Quinolones C) Metronidazole D) All of Above

11) When should a patient with acute pancreatitis be started on a diet?

A) Within 12 hrs B) 12- 24 hrs C) After 24 hrs D) As soon as the patient can tolerate

12) Which route is preferred for nutrition in acute pancreatitis?

A) Parenteral B) Enteral C) Neither

13) Which enteral feeding method is preferred in uncomplicated acute pancreatitis?

A) Nasogastric delivery B) Naso-jejunal delivery C) Either A and B D) TPN

14) When should patients with mild gallstone pancreatitis be considered for cholecystectomy?

A) On admission B) Within 12-24 hrs C) Before discharge from hospital

D) In 4-6 weeks post-discharge

15) Which parameter warrants acute intervention in patients with asymptomatic pseudocyst and pancreatic/extra-pancreatic necrosis?

A) Large size B) Location C) Extent D) Abdominal pain E) None of the above 16) Ideally, in stable patients with symptomatic necrosis in acute pancreatitis, for how long should drainage (surgical, radiological and/or endoscopic) be delayed?

A) Immediately B) Within 24-48 hours C) Within 1 week D) After 4-6 weeks
